# Identifying opportunities for diagnostic stewardship in UTI testing in pediatrics

**DOI:** 10.1017/ash.2023.307

**Published:** 2023-09-29

**Authors:** Karen Acker, Michael Alfonzo, Tess Gray, Taylor Dempsey, Lisa Saiman, Lars Westblade, Sabrina Racine-Brzostek, Nicole Gerber

## Abstract

**Background:** Reflexive urine culturing, a strategy wherein urine cultures are only performed on samples with pyuria, is increasingly being used to reduce unnecessary urine cultures, healthcare costs, and inappropriate antibiotics. To support implementation of a reflexive urine-culture order for pediatric patients aged <18 years, we assessed the proportion of urine cultures that would be avoided with reflexive urine culturing, and we calculated the sensitivity and negative predictive value (NPV) of the ≥10 white blood cells (WBC) per high-powered field (HPF) threshold for diagnosing urinary tract infections (UTI) in patients aged <18 years who presented to the pediatric emergency department (ED). **Methods:** A retrospective review of patients <18 years with a urine culture performed from January to May 2022 in an urban, tertiary-care, pediatric ED was performed. A positive urine culture was defined as ≥50,000 CFU/mL for catheterized specimens and ≥100,000 CFU/mL for clean-catch or unspecified specimens. Pyuria was defined as ≥10 WBC/HPF. ‘True UTI’ was defined as a positive urine culture with a consistent clinical presentation (eg, fever or dysuria). Sensitivity, specificity, and NPV were calculated using the pyuria threshold of ≥10WBC/HPF compared to the gold standard of a ‘true UTI.’ **Results:** During the study period, 658 patients aged <18 years had urine cultures sent, of which 46 (7%) were positive. In all, 407 urine cultures (61.9%) were obtained by clean catch, 233 (35.4%) were obtained by urethral catheterization, 2 (0.3%) were obtained by Foley catheter, and 16 (2.4%) were unspecified. Among the 46 positive cultures, 32 (69.6%) had ≥10 WBC/HPF, and 55 (9.0%) of 612 negative cultures had ≥10 WBC/HPF. Of the 14 patients with positive urine cultures without pyuria, 8 had a contaminated sample or asymptomatic bacteriuria, 3 had urologic abnormalities, and 3 were infants aged <3 months. Of the 14 patients, 3 (21.4%) had a consistent clinical presentation for UTI and were treated with antibiotics: 2 were infants aged <3 months and 1 had urologic abnormalities. Using the ≥10 WBC/HPF threshold compared to ‘true UTI,’ sensitivity was 91.4%, specificity was 91.5%, positive predictive value was 36%, and NPV was 99.5%. Sensitivity and NPV increased to 100% when infants aged <3 months and urologic patients with positive urine culture were excluded. We estimated a cost saving of ~$200,000 had reflexive testing been in place. **Conclusions:** A reflexive urine culture for specimens with ≥10 WBC/HPF would have reduced the number of urine cultures substantially because 571 (86.8%) of 658 urine cultures would not have been performed. To prevent missed diagnoses of UTI, infants aged <3 months and children with urologic abnormalities should be excluded from this diagnostic stewardship intervention.

**Disclosures:** None

